# The Hydroethanolic Stem Bark Extract of *Tieghemella heckelii* (A.Chev.) Pierre ex Dubard (Sapotaceae) Produced N-Methyl-D-Aspartate (NMDA) Receptor-Dependent Analgesia and Attenuates Acute Inflammatory Pain via Disruption of Oxidative Stress

**DOI:** 10.1155/2021/3466757

**Published:** 2021-08-09

**Authors:** Emmanuel K. Kumatia, Regina Appiah-Opong

**Affiliations:** ^1^Centre for Plant Medicine Research, Department of Phytochemistry, Mampong-Akwapim, Ghana; ^2^University of Ghana, Noguchi Memorial Institute for Medical Research, Department of Clinical Pathology, Accra, Ghana

## Abstract

**Background:**

*Tieghemella heckelii* stem bark is used in African traditional medicine to treat inflammatory pain conditions. However, these biological actions of the plant have not been proven. This study investigates the phytochemical composition and the mechanisms of analgesic and anti-inflammatory actions of the hydroethanolic stem bark extract of *T. heckelii* (THBE).

**Methods:**

Phytochemical composition of THBE was investigated using qualitative and quantitative phytochemical analyses. Anti-inflammatory activity was evaluated using the carrageenan-induced paw oedema assay. Analgesic activity was evaluated using hot plate and acetic acid-induced writhing assays. Mechanism of analgesic action was determined using pharmacological antagonist such as naloxone, atropine, flumazenil, nifedipine, or ketamine. Test agents were administered orally as follows: Tween 80 (5%) (control), diclofenac sodium (DS) 10/tramadol 9 mg/kg (standard), or THBE 10, 100, and 450 mg/kg. Glutathione peroxidase (GPx), superoxide dismutase (SOD), and lipid peroxidation levels were also measured.

**Results:**

THBE which contained 58.45% saponins, 229.04 ± 0.049 GAE mg/g phenolic compounds,and 0.482 ± 0.0028 QE mg/g flavonoids produced (*p* < 0.5) anti-inflammatory effect of 56.22% and analgesia of 330 ± 72% and 50.4% in the hot plate and writhing assays, respectively, at 10 mg/kg and inhibited oxidative stress by GPx and SOD elevation in rats during inflammation. Ketamine significantly blocked the analgesia of THBE, indicating NMDA receptor-dependent analgesic action. Whereas, naloxone, atropine, nifedipine, and flumazenil could not antagonize the analgesic action of THBE.

**Conclusion:**

These results show that THBE produced potent anti-inflammatory effect via disruption of oxidative stress and also generated NMDA receptor-dependent analgesia.

## 1. Introduction

Pain is a protective mechanism which warns the body against possible or actual tissue damage when there is a noxious stimulus, physical injury, inflammatory state, or disease. The most prevalent cause for which people seek medical treatment is acute pain which manifests in various forms such as headache, gastrointestinal, musculoskeletal, or chest pains in addition to pain due to injuries, e.g., sprains, lacerations, and fractures [1, 2]. Untreated pain can cause physical damage to body parts or lead to chronic pain which may produce long-term structural and/or genomic alteration in the nervous system [3].

Moreover, inflammation, which is a primary cause of pain, involves a series of well-coordinated biological processes in reaction to obnoxious stimuli in an attempt to get rid of the stimulus and repair the injured part which produces inflammatory pain as a result of direct stimulation of sensory neurons by inflammatory mediators and byproducts of broken-down tissues [4–6].

Plants have been employed by mankind to treat pain and other diseases for several millennia. In addition, some notable analgesics such as aspirin and morphine were derived from medicinal plants. Hence, the need to scientifically explore folkloric medicinal plants with analgesic properties in order to develop them into alternative painkillers. *Tieghemella heckelii* also known as Baku or Cherry Mahogany is a timber tree in the Sapotaceae family. The seed of the plant is used to treat hernia [7]. The young bud is employed against snake bites, and the stem bark is used to treat blennorrhoea and toothache [8]. Few biological activities such as antibacterial, cytotoxicity, and anti-HIV inhibitory effects of the plant have been reported [9, 10]. Although *T. heckelii* is used to treat blennorrhoea and toothache, which is indicative of its anti-inflammatory and analgesic properties, these activities of the plant have not been evaluated. This study, therefore, sought to investigate the mechanism of analgesic and anti-inflammatory actions of the standardized hydroethanolic stem bark extract of *T. heckelii*.

## 2. Materials and Methods

### 2.1. Chemicals and Other Reagents

Carrageenan, diclofenac sodium salt (DS), naloxone, and acetic acid were procured from Sigma Chemical Co. (St. Louis, USA). Tramadol was purchased from Bristol Laboratories Ltd., Luton, England. Normal saline was supplied by Otsuka Pharmaceuticals India Private Limited (Vasana-Chakawadi, India). Flumazenil injection was purchased from Hameln Pharma Plus, GMBH, Germany. Ketamine hydrochloride injection was also purchased from Psychotropics India Ltd., Haridwar-249403 (Uttarakhand), India. Nifedipine was obtained from Denk Pharma GMBH and Co., KG, Germany. Sodium chloride was supplied by Timstar Laboratory Suppliers Ltd., Cheshire, England. Extraction was carried out using food-grade ethanol and distilled water.

### 2.2. Collection and Extraction of *T. heckelii*

The plant was identified and collected from the Bobiri Forest Reserve (Kumasi, Ashanti Region, Ghana) by Mr. Jonathan Dabo, a botanist from the Forestry Research Institute of Ghana (FORIG), Kumasi. The stem bark was sun dried for eight days and (voucher specimen number FORIG 0013) deposited at the herbarium of FORIG. It was milled into course powder. Thereafter, 200 g of the powder was extracted twice with 70% ethanol (2 L) at room temperature for four days each filtered and combined. The ethanol was evaporated from the extract in a rotary evaporator (Eyeler N1110, Tokyo-Japan). The aqueous portion was lyophilized into a powder and coded THBE.

### 2.3. Experimental Animals

SDR rats, C57BL/6, and ICR mice were supplied by the Animal Experimentation Unit (AEU) of CPMR, Mampong-Akuapim, Ghana. The rodents were housed under standard conditions and fed on powered chow and sterilized water.

### 2.4. Determination of the Classes of Phytochemical Ingredients Present in THBE

Approximately, 150 mL of the extract was evaporated to about 40 mL and then mixed with 60 mL of distilled water and allowed to cool. The aqueous sample was then tested for the presence or absence of diverse groups of phytochemical ingredients [11].

### 2.5. Acute Toxicity Test of THBE

Acute toxicity effect of THBE at 2500 mg/kg p.o. reconstituted in 10 mL of water was assessed in a single set of female SDR rats (100–135 g; *N* = 4) with some modifications as reported earlier [12]. The rats were observed for indicators of toxicity such as change in motor activity, eye color, salivation, lachrymation, coma, and/or eventual death in 14 days.

### 2.6. Determination of Total Flavonoids Content in THBE

The quantity of flavonoids in THBE was determined using the aluminum colorimetric method with some modifications [13]. A calibration curve was plotted for quercetin from which the quantity of flavonoids in THBE was determined as milligram of quercetin equivalents (mg of QE) per gram of extract. Water served as blank.

### 2.7. Determination of Total Phenolic Content in THBE

The quantity of phenolic compounds in THBE was determined using the Folin-Ciocalteau method [14]. The test was carried out in triplicates. A standard calibration curve was generated using gallic acid and the phenolic content in THBE determined from the standard curve as microgram of gallic acid equivalent (GAE) per milligram of dry sample.

### 2.8. In Vitro Antioxidant Activity Test

The in vitro free radical scavenging capacity of the extract was determined using the 2, 2-diphenyl-1-picrylhydrazyl (DPPH) method [15]. Ascorbic acid was used as a standard. Antioxidant activity (%) was calculated using the following formula:

(1) $$\begin{array}{c} \%\text{ antioxidant}=\left(\frac{\text{control OD}-\text{sample OD}}{\text{control OD}}\right)\;x\;100\%, \end{array}$$

where OD represents the optical density.

The concentration of the test sample which produced the 50% antioxidant effect was taken to be the half-maximal inhibitory concentration (IC_50_).

### 2.9. Evaluation of Anti-Inflammatory Activity of THBE

The acute anti-inflammatory effect of THBE was evaluated using the carrageenan-induced paw oedema assay [16]. Five (5) groups of female SDR rats (197–212 g; *N* = 4) were given 1 mL of 5% Tween 80 solution as vehicle control (group 1); the standard drug DS 10 mg/kg p.o. (group 2) and THBE 10, 100, and 450 mg/kg p.o., respectively (groups 3–5). Baseline paw volumes (Vo) were determined as the average of two measurements with a plethysmometer (UGO Balise, 7140) prior to any treatment. Inflammation was induced by subplantar injection of 0.1 mL of 1% w/v carrageenan in normal saline into the right hind paw of each rat. The paw volume was then measured at 1, 2, 3, and 4 h. The overall oedema response was calculated as the net area under the time course curves (AUC). The anti-inflammatory activity was also calculated as

(2) $$\begin{array}{c} \text{anti}‐\text{inflammatory activity}=\frac{\left(\text{mean AUC of CT}-\text{ mean AUC of TG}\right)}{\left(\text{mean AUC of CT}\right)}\;x\;100\%, \end{array}$$

where CT is the control group and TC is the treated group.

### 2.10. Analgesic Assays

#### 2.10.1. Hot Plate Assay

In the first analgesic activity assay, the mouse hot plate assay was used [17]. In summary, twenty-eight (28) male C57BL/6 mice were divided into seven groups (7) of four (4) mice each. Latency of each mouse was determined by stationing it on an electric hot plate (Ugo Basile hot/cold plate 35100) sustained at 55 ± 0.5°C. The time taken by the mouse to react to the thermal stimulus by shaking, licking, lifting, or stamping any of its hind limbs or to jump was recorded as reaction time or latency time. Baseline latency (To) was obtained for each mouse as the average of two (2) pretreatment measurements before administration of any test agent. The mice selected for the test have a baseline latency range of 3.5–9.0 s. Group 1 was given 5% Tween 80 in distilled water as the vehicle control. Tramadol 6 mg/kg p.o. served as the standard drug (group 2). Groups 4–5 also received THBE at 10, 100, and 450 mg/kg p.o., respectively. Thereafter, the latency of each mouse was measured at 1, 2, 3, and 4 h (Tt). The analgesic activity was calculated as percentage of pain threshold inhibition (%PTI) as [18]

(3) $$\begin{array}{c} \%\text{PTI }=\frac{\text{Tt }–\text{ To}}{To}\;x\;100. \end{array}$$

#### 2.10.2. Analgesic Activity of THBE in Acetic Acid-Induced Writhing Assay

The analgesic activity of THBE was also evaluated using the acetic acid-induced writhing assay [19]. In this assay, five (5) groups of male ICR mice (31–37 g), which consist of four (4) mice each, were administered 5% Tween 80 in distilled water as control (group 1), 10 mg/kg p.o. of DS as standard drug (group 2), and THBE 10, 100, and 450 mg/kg p.o. (groups 3–5). Acetic acid (1%) was intraperitoneally administered at 1 mL/10 g per bodyweight of each mouse 45 min afteradministration of the test agents. The mice were then transferred into individual transparent plastic cages. The writhing movement of each mouse was counted for 20 min. Analgesic activity was calculated using the following formula:

(4) $$\begin{array}{c} \text{analgesic activity }=\frac{\text{MRc}-\text{ MRt }}{\text{MRc}}\;x\;100\%, \end{array}$$

where MRc represents the mean writhing count of the control and MRt represents the mean writhing count of THBE or the DS treated group.

### 2.11. Evaluation of Mechanism of Analgesic Action

#### 2.11.1. Hot Plate Test

The effect of naloxone, a nonselective opioid antagonist, on the analgesic action of THBE was determined in the hot plate test according to Kumatia et al. [20]. Three (3) groups of C57BL/6 mice (*N* = 4) were used. Group 1 received 5% Tween 80 solution as vehicle control. Group 2 received THBE 100 mg/kg p.o. in 5% Tween 80 solution. Group 3 also received THBE 100 mg/kg p.o. followed by naloxone 2 mg/kg (i.p.) in distilled water at 1 mL/10 g after 35 min. Analgesic activity was then determined using the hot plate test.

#### 2.11.2. Acetic Acid-Induced Writhing Test

The effects of other antagonists such as atropine, flumazenil, nifedipine, and ketamine on the analgesic action of THBE were determined using seven groups of male ICR mice (*N* = 4) in the acetic acid-induced writhing assay. Group 1 (control) received 5% Tween 80 solution. Group 2 received THBE 100 mg/kg p.o. Groups 3–6 also received THBE 100 mg/kg p.o. each. After 35 min, atropine (10 mg/kg i.p. in distilled water), flumazenil (10 mg/kg i.p.), nifedipine (30 mg/kg p.o. in distilled water), and ketamine (30 mg/kg i.p. in distilled water) at 1 mL/10 g were administered, respectively, to groups 3, 4, 5, and 6. Analgesic activity was then determined for each group using the acetic acid-induced writhing assay 10 min after administration of each antagonist.

### 2.12. Effect of THBE Treatment on Oxidative Stress

#### 2.12.1. Preparation of Liver Samples for Determination of Oxidative Stress Indices

After the termination of the carrageenan-induced inflammation in the rat paws assay at 4 h, the rats were sacrificed by cervical displacement and instantly dissected. The livers were removed and processed to obtain a supernatant fluid [21].

#### 2.12.2. Determination of Glutathione Peroxidase (GPx) Action

The amount of GPx produced was determined by reaction of 5, 5-dithiobis (2-nitrobenzoic acid) (DTNB) with reduced glutathione to produce a yellow chromogen whose concentration was measured with a spectrophotometer at an absorbance of 405 nm [22].

#### 2.12.3. Determination of Superoxide Dismutase (SOD) Action of THBE

SOD action of the extract in the liver sample was determined as described [23]. Quercetin was employed as the substrate. SOD activity was calculated as the quantity of the enzyme which inhibits oxidation of quercetin by 50%.

#### 2.12.4. Determination of Lipid Peroxidation Action of THBE

Lipid peroxidation action of THBE was measured in the thiobarbituric acid reactive substances (TBARS) test [21]. The level of lipid peroxidation was calculated as thiobarbituric acid reactive substances (TBARS) expressed as malondialdehyde (MDA) formation in moles/mg of tissue.

### 2.13. Statistical Analysis

Data were analyzed with GraphPad Prism 5 statistical software. Results were deemed statistically significant when the difference between the control and test group means is < 0.05 (*p* < 0.05).

## 3. Results

### 3.1. Yield and Nature of the Extract

The 70% hydroethanolic extract of the 200 g dried stem bark of *T. heckelii* produced 43.76 g of reddish brown hygroscopic solid (coded THBE) which translated into a yield of 21.88% w/w.

### 3.2. Classes of Phytochemical Constitutes Present in THBE

The result of the phytochemical screening of the extract is given in Table 1.

### 3.3. Quantitative Phytochemical Results and Antioxidant Activity of THBE in the DPPH Assay

The quantities of various phytochemicals and the results of the antioxidant activity of the extract in the DPPH assay are given in Table 2. *R*^2^ of 0.9177 and 0.8200 were obtained for THBE and ascorbic acid, respectively.

### 3.4. Toxicity or Safety of THBE Administration in Acute Conditions

The extract administered at 2500 mg/kg p.o. did not produce any observable sign of toxicity such as change in motor activity, eye color, salivation, lachrymation, coma, or eventual death within the 14 days observation period in female SDR rats.

### 3.5. Analgesic Effect of THBE

#### 3.5.1. Analgesic Effect of THBE on Thermal-Induced Pain

**Figure 1:**
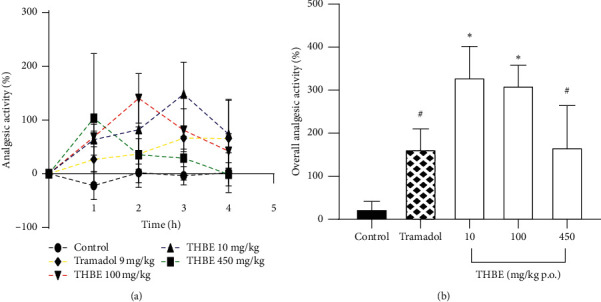
Analgesic effect of THBE in C57BL/6 mice in the hot plate test on the time course curve (a) and overall analgesic activity calculated as area under the time course curve (b).  ^*∗*^*P* < 0.05, ^#^*p* > 0.05 compared to the (untreated) control mice.

The analgesic effect produced by THBE in the hot plate test is shown in Figure 1 as time course curve and as overall analgesic activity calculated as AUC of the time course curve.

THBE inverse dose-dependently increased the latency of C57BL/6 mice to react to thermal-induced nociceptive pain in the hot plate assay. This culminated in significant (*p* < 0.05) analgesic activity of 330 ± 72 and 311.2 ± 46.81% for THBE 10 and 100, respectively. Tramadol 9 mg/kg p.o. and THBE 450 mg/kg p.o. also produced (*p* > 0.05) analgesic activity of 163.1 ± 47.21 and 167.6 ± 96.92%, respectively.

#### 3.5.2. Analgesic Effect of THBE on Chemically Induced Pain

**Figure 2:**
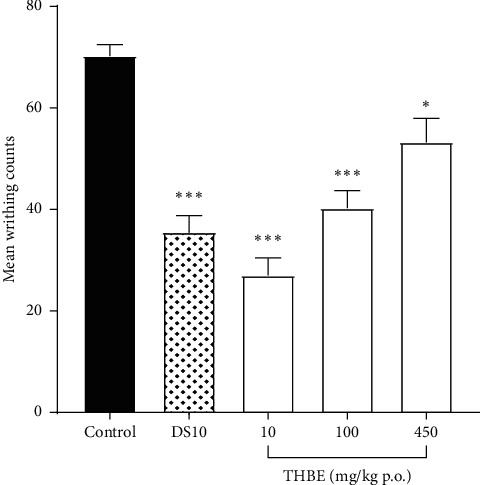
Effect of THBE or DS on inhibition of chemically induced pain in the acetic acid-induced writhing test.  ^*∗*^*P* < 0.05,  ^*∗∗∗*^*p* < 0.001 compared to the (untreated) control mice.

The effect of THBE or DS on writhing counts in mice is shown in Figure 2.

THBE and DS significantly (*p* < 0.05) inhibited the mean writhing counts at all dose levels compared to the control mice. The highest analgesic activity of 50.4% was observed at THBE 10 mg/kg p.o. The analgesic activities of THBE at 100 and 450 mg/kg p.o. or DS 10 mg/kg p.o. are 30.0, 23.9, and 49.5%, respectively.

### 3.6. Effect of THBE on Carrageenan-Induced Acute Inflammatory Response

**Figure 3:**
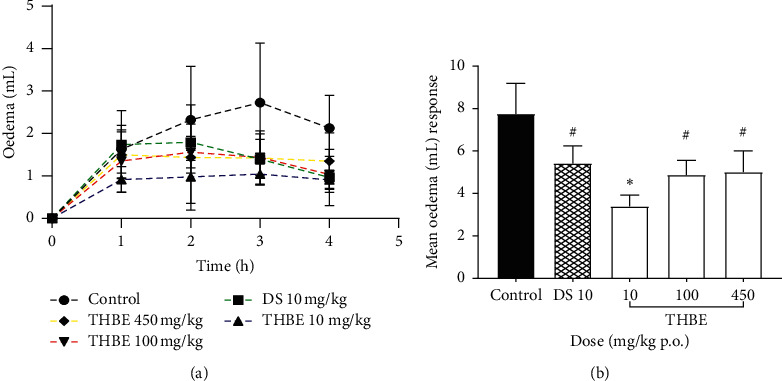
Anti-inflammatory effect of THBE and DS on carrageenan-induced acute inflammation in rats' paws on time course curve (a) and as mean oedema response (b).  ^*∗*^*P* < 0.05, ^#^*p* > 0.05 compared to the (untreated) control rats.

The effect of oral administration of THBE on carrageenan-induced inflammation in rats is shown in Figure 3 on the time course curve and as mean oedema response.

THBE produced inverse dose-dependent reduction in oedema/inflammation in the treated rats with corresponding anti-inflammatory activity of 56.22, 37.11 and 35.41% respectively. The anti-inflammatory activity of DS was 30.13%. The anti-inflammatory activity of THBE 10 mg/kg p.o. was statistically significant (*p* < 0.05) but DS 10 mg/kg p.o. and THBE 100 or 450 mg/kg p.o. produced statistically insignificant (*p* > 0.05) anti-inflammatory activity as a result of the high S.E.M they recorded.

### 3.7. Effect of THBE on Oxidative Stress Indices

**Figure 4:**
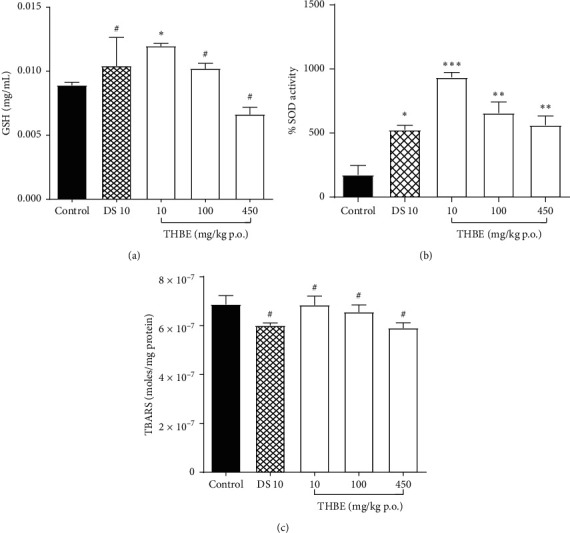
Effect of THBE on GPx (a), SOD (b), and lipid peroxidation (c) in rats' liver during inflammation.  ^*∗*^*P* < 0.05,  ^*∗∗*^*p* < 0.05,  ^*∗∗∗*^*p* < 0.05, ^#^*p* > 0.05 compared to the (untreated) control mice.

Figures 4(e)–(g) show the effect of THBE and DS on hepatic oxidative stress indices such as GPx, SOD, and lipid peroxidation in rats' liver during carrageenan-induced inflammation.

#### 3.7.1. Glutathione Peroxidase (GPx)

The extract inverse dose-dependently increased hepatic GPx levels in response to carrageenan-induced acute inflammation in rats' paw. Increased GPx activity was statistically significant (*p* < 0.05) only for THBE 10 mg/kg p.o (Figure 4(a)).

#### 3.7.2. Superoxide Dismutase (SOD)

Also, THBE 10–450 mg/kg p.o. and DS 10 mg/kg p.o. significantly (*p* < 0.05) and inverse dose-dependently elevated hepatic SOD activity in rats during acute inflammation (Figure 4(b)).

#### 3.7.3. Lipid Peroxidation

The result shows that THBE and DS were not able to significantly (*p* > 0.05) lower the production of TBARS in carrageenan-treated rats after 4 hours of administration (Figure 4(c)).

### 3.8. Mechanism of Analgesic Action of THBE

**Figure 5:**
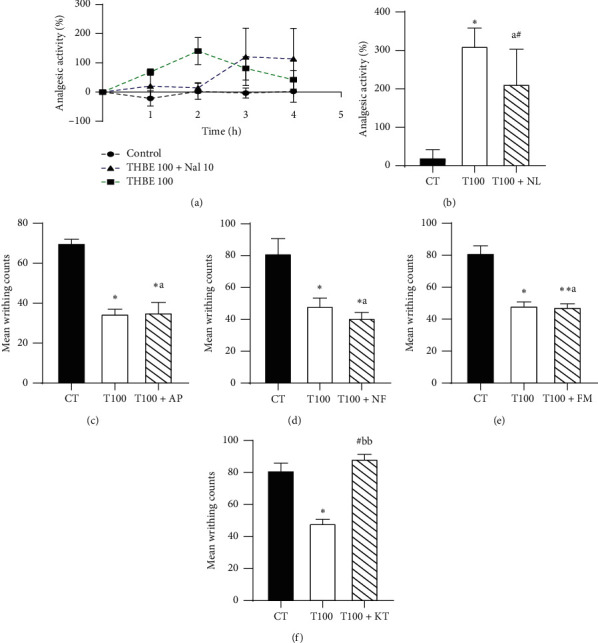
Effect of naloxone, NL (a), (b), atropine, AT (c), nifedipine, NF (d), flumazenil, FM (e), and ketamine, KT (f) on the analgesia action of THBE.  ^*∗*^*P* < 0.05,  ^*∗∗*^*p* < 0.05,  ^*∗∗∗*^*p* < 0.05, ^#^*p* > 0.05 compared to the (untreated) control mice. ^b^^b^*P* < 0.01, ^a^*P* > 0.05 compared to the THBE 100 mg/kg p.o. treated mice. T100, THBE 100 mg/kg p.o.; CT, control group.

The results of the effect of the various pharmacological antagonists on the analgesic activity of the extract are shown in Figures 5(a)–5(f).

Co-administration of THBE 100 mg/kg p.o. and naloxone in the hot plate test did not reduce the latency of mice to react to nociceptive pain in the hot plate assay (Figures 5(a) and 5(b)). Furthermore, coadministration of THBE 100 mg/kg p.o. and atropine, nifedipine, flumazenil, or ketamine in the writhing test also produced effective analgesia by inhibition of writhing movement in mice except for the ketamine treatment group. Administration of THBE 100 mg/kg p.o. alone also produced effective (*p* < 0.05) analgesia in the hot plate test and in the acetic acid-induced writhing test (Figures 5(c)–5(f)).

## 4. Discussion

The anti-inflammatory test results indicate that the extract produced significant (*p* < 0.05) anti-inflammatory action against carrageenan-induced acute inflammatory pain, such that the highest anti-inflammatory response (56.22%) produced by the extract (THBE 10 mg/kg p.o.) was almost 2-fold (1.9) greater than that of the standard NSAID drug (DS 10 mg/kg p.o.), 30.13%. This indicates that the extract is a potent anti-inflammatory agent against acute inflammatory pain.

Previous studies reported that acute inflammation generates explicit pain through the direct stimulation of sensory neurons with inflammatory mediators such as prostaglandins, substance P, bradykinin, histamine, serotonin, and cytokines released by broken-down tissues which transmit and amplifies the pain signal leading to central sensitization and clinical pain states [4–6]. The therapeutic mechanism of action of analgesics is therefore tailored towards the disruption of inflammatory mediators, neurotransmitters, and/or neuropeptides involved in the processes of pain transmission and perception with NSAIDs attacking the inflammation reaction that produces sensitization by inhibition of cyclooxygenase (COX) and hence reduced prostaglandins (PGs) production [24, 25].

The result shows that the extract possessed significant (*p* < 0.05) anti-inflammatory action against carrageenan-induced acute inflammation. This indicates that the extract might promote inhibition of COX and PGs syntheses which resulted in decreased sensitization of nociceptors and clinical pain state.

Inflammation is an immune reaction which amplifies oxygen consumption of immune cells (e.g., mast cells and leukocytes) and leads to increased production and buildup of reactive oxygen species (ROS) [26]. Excess ROS in the body leads to oxidative stress which occurs when the body's inborn antioxidant defense grid is overpowered by excess ROS. Oxidative stress has been reported to be the basic mechanism for diverse pain-related symptoms [27]. ROS production was also reported in the carrageenan-induced paw oedema assay [28]. Besides, the liver is a major organ in the body which performs cellular homeostasis and produce various endogenous antioxidant enzymes to counter the action of ROS. Hence, the overall oxidative state of the body may be indicated by the levels of antioxidant enzymes in the liver. SOD and GPx are first-line antioxidant enzymes which inhibit ROS and other free radicals productions [29]. GPx also mop up active ROS and thus prevent chain initiation and proliferation reactions [30]. The results show that the extract significantly (*p* < 0.05) elevated GPx and SOD and also produce high antioxidant activity with IC_50_ of 0.012 mg/mL (the same IC_50_ obtained for ascorbic acid) in the DPPH assay. This indicates that the ameliorating effect of THBE against inflammatory pain is also due to its high in vivo and in vitro antioxidant action which reverses oxidative stress by inhibition of ROS production and mopping up of active free radicals.

The extract and DS were also accessed for their protective effect against the destruction of cell membrane lipids, distortion of structure, and functions of cell membrane and destruction of DNA and enzymes such as polymerases which repairs damaged DNA during oxidative stress in the lipid peroxidation assay [30]. The result shows that the extract and DS could not offer significant (*p* > 0.05) protection against lipid peroxidation during acute inflammation.

The hot plate test is an acute pain model in which thermal stimulus causes injury and inflammation to tissues resulting in secretion of peripheral mediators such as potassium ions, hydrogen ions, adenosine triphosphate (ATP), and glutamate from the cytosol [31, 32]. The result shows that the extract produced significantly (*p* < 0.05) high analgesic effect in the hot plate test. This indicates that the extract is able to ameliorate thermal-induced acute pain and inflammation via inhibition of release of peripheral mediators.

The acetic acid-induced writhing assay is used to evaluate the analgesic and anti-inflammatory actions of substances because injection of algogens such as acetic acid into the abdominal cavity of mice produces inflammatory reaction with consequent activation of nociceptors which results in acute inflammation and pain in the abdominal region [33]. THBE produced significant (*p* < 0.05) analgesia by reduction of writhing count in the acetic acid-induced writhing assay. This indicates that THBE possessed both analgesic and anti-inflammatory actions. Also, THBE produced significant anti-inflammatory action in the carrageenan-induced inflammatory assay. THBE may, therefore, have a similar mechanism of action as NSAIDS which produce both anti-inflammatory and analgesic actions by inhibition of COX leading to reduction in PGs synthesis.

In the hot plate and acetic acid-induced writing assays, the analgesic effect of THBE was inverse dose-dependent. This indicates that at higher doses, the extract might lose its analgesic activity or produces hyperalgesic effects at the pain receptors through antagonistic action. This mode of action of THBE is similar to those of naloxone and ketamine which are used as analgesic drugs at low doses and as antagonist at higher doses.

It has been reported that analgesic drugs such as opioids, antiepileptics, local anesthetics and other analgesics block, or modulate channels, hence preventing the production or transmission of nerve impulses in order to produce analgesia [2]. Hence, in order to elucidate the mechanism of action of THBE as analgesic agent, a series of assays were conducted in the hot plate and the acetic acid-induced writhing assays by employing various receptor antagonists to block the antinociceptive action of the extract in order to characterize the receptors/channels involved in its analgesic action. The hot plate assay is used to evaluate the analgesic action of centrally active analgesics. It has also been reported that opioid receptors which are made up of three (3) subtypes (mu, kappa and delta) are mainly found in the central nervous system (CNS) and also in peripheral nociceptors [34]. The nonselective opioid receptor antagonist, naloxone, was therefore co-administered with the extract to block the opioid receptor in the CNS. However, naloxone did not antagonize the analgesic action of the extract. Hence, a different receptor but not an opioid receptor was involved in the analgesic action of THBE in the hot plate test.

The acetic acid-induced writhing assay was reported to be a nonselective antinociceptive model since it acts by secondary activation of the release of endogenous mediators which stimulates nociceptive neurons which are receptive to NSAIDs, opioids, and other centrally [35] and peripheral acting drugs. Hence, the effect of atropine (the cholinergic receptor antagonist), nifedipine (calcium channel blocker), flumazenil (the selective gamma-aminobutyric acid A (GABA_A_) receptor antagonist), and ketamine (the uncompetitive *N-*methyl-D-aspartate receptor (NMDA) antagonist) were studied on the analgesic action of THBE in the writhing assay. The results show that only ketamine completely blocked the analgesic action of THBE in the acetic acid-induced writhing assay. This indicates the involvement of the *N-*methyl-D-aspartate receptor (NMDA) in the analgesic action of THBE.

The phytochemical analysis showed that flavonoids, phenolic compounds, saponins, antracenosides, and phytosterols were present in THBE. The quantitative test also shows that the extract contains high quantities (229.04 ± 0.049 GAE mg/g) of phenolic compounds in addition to 0.482 ± 0.0028 QE mg/g of flavonoids. Phenolic compounds which includes flavonoids easily donate their phenolic hydrogen atom (H^+^) to react with chain carrying radicals (ROO ^*∗*^) in ROS and RNS (reactive nitrogen species) in an end reaction which terminates the cycle of production of new radicals and also chelate metal ions involved in the production of free radicals and hence preventing oxidative stress [36, 37] and treating oxidative stress-related diseases such as inflammation and pain. Therefore, the profound anti-inflammatory and analgesic action of THBE could be attributed in part to its flavonoid and high phenolic constituents.

Moreover, quercetin, rutin, and apigenin which are both flavonoids and phenolic compounds were shown to produce analgesic and anti-inflammatory properties by reduction of TNF-*α*, IL-1*β*, IL-6, and NO generation [38–41]. Other reports indicated that quercetin produce anti-inflammatory action in mouse models of colitis, periodontitis, cancer pain, and acute pain via exertion of the inhibitory effect on neutrophil recruitments, oxidative stress, and COX-2 [42–44]. Also, saponin containing plant such as *Stauntonia chinensis* has been reported to demonstrate significant analgesia in the mouse hot plate, acetic acid-induced writhing, formalin, and capsaicin tests through elevation of the inhibitory synaptic response in cortical neurons [45]. Therefore, the presence of flavonoids, phenolic compounds, and saponins in THBE may contribute to its profound attenuation of acute nociceptive and inflammatory pain in this study.

The quantitative results of the constituents of THBE can also serve as standard parameters for *T. heckelii* stem bark to ensure consistency in biological activity of future samples of *T. heckelii* stem bark [46].

Finally, the result from the acute toxicity test indicates that the extract is relatively safe, since it did not produce any observable sign of toxicity or eventual death within the 14 days observation period in female SDR rats even at a very high dose of 2500 mg/kg p.o.

## 5. Conclusion

In conclusion, the results indicate that 70% hydroethanolic stem bark extract of *T. heckelii* (THBE) is relatively safe and possessed remarkable anti-inflammatory and analgesic actions. The anti-inflammatory action of the extract against acute nociceptive pain may be due to the profound antioxidant capacity of the extract as a free radical scavenging agent which counters the destructive effect of oxidative stress. The analgesic action of THBE was *N-*methyl-D-aspartate receptors (NMDA) dependent. These pharmacological properties of the plant were due to its constituents such as phenolic compounds, flavonoids, saponins, and phytosterols. These results justified the use of the stem bark extract of *T. heckelii* as an analgesic agent in traditional medicine. Further work will be performed to isolate and characterize the chemical ingredients present in the extract and subject them to preclinical analgesic and anti-inflammatory activity tests.

## Figures and Tables

**Table 1:** Phytochemical constituents present or absent in THBE.

Constituents present	Constituents absent
Flavonoids	Alkaloids
Saponins	Antracenosides
Triterpenes	Polyuronoids
Free reducing sugars	Cyanogenic glycosides
Phytosterols	
Phenolic compounds	

**Table 2:** Quantity of some phytochemical in THBE and its antioxidant activity in the DPPH assay.

Constituent	Quantity	DPPH free radical scavenging activity	
Flavonoids	0.482 ± 0.0028 QE mg/g	IC_50_ (THBE)	0.012 mg/mL
Phenolic compounds	229.04 ± 0.049 GAE mg/g	IC_50_ (ascorbic acid)	0.012 mg/mL
Saponins	58.45%	—	—

QT, quercetin equivalent; GAE, gallic acid equivalent.

## Data Availability

The data for this study can be obtained from the corresponding author upon reasonable request.

## Abbreviations

THBE: 70% ethanol extract of *T. heckelii* stem back
NMDA: *N-*Methyl-D-aspartate
DS: Diclofenac sodium salt powder
GPx: Glutathione peroxidase
SOD: Superoxide dismutase
GAE: Gallic acid equivalent
QE: Quercetin equivalent
TBARS: Thiobarbituric acid reactive substances
MDA: Malondialdehyde
NL: Naloxone
AT: Atropine
NF: Nifedipine
FM: Flumazenil
KT: Ketamine
CT: Control group.
